# Intracerebral Transplants of GMP-Grade Human Umbilical Cord-Derived Mesenchymal Stromal Cells Effectively Treat Subacute-Phase Ischemic Stroke in a Rodent Model

**DOI:** 10.3389/fncel.2020.546659

**Published:** 2020-09-25

**Authors:** Jeong-Eun Noh, Seung-Hun Oh, In-Hyun Park, Jihwan Song

**Affiliations:** ^1^Department of Biomedical Science, CHA Stem Cell Institute, CHA University, Seongnam-si, South Korea; ^2^Department of Neurology, CHA Bundang Medical Center, CHA University, Seongnam-si, South Korea; ^3^Department of Genetics, Yale Stem Cell Center, Yale School of Medicine, New Haven, CT, United States; ^4^iPS Bio, Inc., Seongnam-si, South Korea

**Keywords:** stroke, intracerebral transplantation, umbilical cord (UC), mesenchyaml stem cells, GMP (good manufacturing process)

## Abstract

In subacute and chronic phases of the stroke, there are no therapeutics available at present to promote functional recovery. Human umbilical cord-derived mesenchymal stromal cells (hUC-MSCs) are one of the candidate cell types for treating subacute-phase stroke. The benefits of cell-based therapy largely depend on the migratory capacity of products administered, as well as their potential for engraftment in targeted tissues and paracrine activities. Timing and delivery modes may also influence the outcomes of stem-cell therapy. Still, the functional recuperative effects of differing hUC-MSC delivery modes, about cell replacement and cell-to-cell paracrine activity levels, have yet to be clarified in subacute phases of stroke.This study was conducted to compare the therapeutic effects of various delivery routes when administering Good Manufacturing Practice (GMP)-grade hUC-MSCs in a rodent model of subacute-phase stroke. Cell aliquots (1 × 10^6^) were given to rats as intravenous (IV) injections or intracerebral (IC) transplants 1 week after middle cerebral artery occlusion (MCAo). Transplanted rats were examined up to 7 weeks later using various behavioral tests and immunohistochemical analyses. Most IC-transplanted cells survived for short periods (i.e., <4 weeks after receipt) and gradually disappeared, whereas IV-injected cells were undetectable in the brain at the same time points (i.e., 3 days, 4 weeks, or 7 weeks after injection). Although short-lived, IC-transplanted cells effectively improved behavioral deficits, serving to reduce infarct volumes and glial scar formation, increase subventricular counts of proliferating neuroblasts, and promote cerebrovascular ingrowth in ischemic penumbra regions. IV injection, however, failed to improve behavioral function or histologic parameters during the same 7-week time frame. These findings overall suggest that IC transplantation is preferable to IV injection for delivery of hUC-MSCs during subacute phases of stroke.

## Introduction

Severe brain damage inflicted by strokes leads to long-term disability or even death, and the need for more effective therapies is dire. Ischemic stroke is triggered through multiple mechanisms, involving a cascade of pathophysiologic events that include excitotoxicity, oxidative stress, apoptosis, and inflammation. Spontaneous neurogenesis, astrocytic activation, and angiogenesis may occur in response (Jauch et al., 2013; Powers et al., 2015). In recent years, experimental and clinical trial data have indicated that cell-based therapies of various sorts, incorporating mesenchymal stromal cells (MSCs), neural stem cells, or induced pluripotent stem cells, may offer real opportunities for cell replacement and endogenous brain repair in patients with strokes (Liu et al., 2014).

MSCs have emerged as the leading cell source in the treatment of stroke. They secrete multiple trophic factors for endogenous brain repair [vascular endothelial growth factor (VEGF) and hepatocyte growth factor (HGF); Ranganath et al., 2012] and strong immune-modulating anti-inflammatory mediators [transforming growth factor-β (TGF-β), interleukin (IL)-10, and indoleamine 2,3-dioxygenase (IDO); Ranganath et al., 2012]; and they are present in abundance, found in bone marrow (Pittenger et al., 1999), adipose tissue (Zuk et al., 2002), umbilical cord (Erices et al., 2000), peripheral blood (Ukai et al., 2007), and dental pulp (Gronthos et al., 2000), as well as a wide range of mesodermal reservoirs that include perivascular sites in the brain (Kang et al., 2010; Paul et al., 2012). MSCs may be rapidly expanded *in vitro*, without undermining their innate pluripotency. They are also relatively immune-privileged, carrying a reduced risk of rejection. Human umbilical cord MSCs (hUC-MSCs) isolated from Wharton’s jelly generally escape immune surveillance due to the absence of the major histocompatibility complex II antigen and co-stimulatory surface antigens CD40, CD80, and CD86 (Weiss et al., 2008; Lalu et al., 2012; Bongso and Fong, 2013). Umbilical cords harbor an auspicious pool of fetal cells that act as multipotent stromal cells (Romanov et al., 2003; Can and Karahuseyinoglu, 2007; Batsali et al., 2013). Unlike MSCs in adult tissues, the proliferative and immunosuppressive capacities of hUC-MSCs are comparatively greater (El Omar et al., 2014).

The therapeutic benefits of cell-based therapies depend on their capacities for cellular migration and the extent to which grafted cells inhabit the targeted tissues (Chrostek et al., 2019). Delivery routes are likewise highly influential in the migration and final destination of transplanted cells, impacting therapeutic efficacy. The best means of stem-cell delivery to promote recovery at sites of ischemic brain damage has thus become a major focus of research. Many investigators have explored the efficacy of various routes, including intravenous (IV; Li et al., 2015), intracerebral (IC; Jin et al., 2005; Denham et al., 2012), intraventricular (Jin et al., 2005), and intrathecal injections by lumbar puncture (Lim et al., 2011). MSCs administered as treatment of stroke typically are delivered by IV injection, which is safe and practical. However, recent clinical trials have shown that IV injection of MSCs produces a modest or little therapeutic effect in patients at subacute phases of stroke (Lee et al., 2010; Hess et al., 2017). Although intraventricular or intranasal transfer are alternate options for stem cell delivery, there have been few reported cases, and standardization of these methods has yet to be optimized (Rodríguez-Frutos et al., 2016). On the other hand, IC delivery may offer an ideal route for stem-cell transplantation to damaged brain areas, despite the rather invasive surgical procedures required. Importantly, the outcomes of IC transplantation seem to surpass those of IV injection in terms of functional recovery (Chrostek et al., 2019).

Another issue for cell therapy is the timing of cell delivery. Because the therapeutic window for thrombolytic therapy is quite narrow in clinical situations, <10% of patients with strokes benefit from thrombolytic therapy (National Institute of Neurological Disorders and Stroke rt-PA Stroke Study Group, 1995). Beyond this time frame, there is otherwise a void in established neuroprotective/neurorestorative treatments that is perhaps best addressed by cell therapy. However, data is scarce on direct comparisons of therapeutic effects by MSC delivery routes in subacute or chronic phases of ischemic stroke.

Herein, we have compared efficacies of IC transplantation and IV injection, using Good Manufacturing Practice (GMP)-grade hUC-MSCs in a rat model of subacute stroke. IC transplantation ultimately proved to be the preferred route, culminating in functional recovery. Our data provide critical insights into optimal modes of stem-cell delivery for future clinical applications of MSCs aimed at subacute or chronic phases of stroke.

## Materials and Methods

### Generation of GMP-Grade hUC-MSCs

This study was authorized by an Institutional Review Board at CHA Bundang Medical Center, permitting the use of human umbilical cord (hUC) samples (IRB no.: BD2013-004D). The investigational protocol fully adhered to institutional guidelines and regulations. Preparation of hUC-MSCs, including their isolation and expansion, was undertaken within a GMP facility and performed in accord with Good Clinical Practice (GCP) guidelines of the Master Cell Bank (MCB) at CHA Bundang Medical Center, as previously described (Oh et al., 2018). A healthy volunteer at the center granted informed consent, releasing an hUC specimen for this purpose. Processing was completed within 24 h.

After removing the umbilical vessels, we sliced Wharton’s jelly into explants of 1–2 cm. The slices were attached and placed on culture plates in 500 ml of α-MEM (Thermo Fisher Scientific, Waltham, MA, USA) supplemented with 50 ml of 10% FBS (Thermo Fisher Scientific, Waltham, MA, USA), 25 ng/ml FGF4 (R&D Systems, Minneapolis, MN, USA), and 0.2 U/ml heparin (Sigma–Aldrich, St. Louis, MO, USA) for incubation, changing the medium every 3 days. After 15 days, the cord fragments were discarded. The cells were subsequently passaged using TrypLE Select (Thermo Fisher Scientific, Waltham, MA, USA) and expanded to the point of sub-confluence (80–90%). They were incubated under hypoxic conditions (3% O_2_, 5% CO_2_) at 37°C, housing hUC-MSCs of passage 2 in the Master Cell Bank (MCB). We then thawed cells for subculture under the same conditions. At passage 4, the cells were stored in the Working Cell Bank (WCB). Again, cells were thawed and further cultured to passage 7, mixing a collection of 2 × 10^7^ cells with 1 ml of Cryostor CS10 (BioLife Solutions, Bothell, WA, USA) in an AT-Closed Vial (Aseptic Technologies, Les Isnes, Belgium). The final product was stored at −35°C. Only hUC-MSCs in passage 7 served in the present study.

To test the eligibility of the final cell product, karyotyping and viral tests were done. Karyotype analysis confirmed a normal human karyotype. Using reverse transcriptase PCR, the absence of viral pathogens (human immunodeficiency virus-1 and 2, cytomegalovirus, hepatitis B virus, hepatitis C virus, human T lymphocytic virus, Epstein-Barr virus, mycoplasma, and syphilis) in cell pellets was ensured. The hUC-MSCs produced also expressed high levels of expected cell surface markers (CD44, CD73, CD90, and CD105), showing extremely low expression levels of hematopoietic stem-cell markers (CD31, CD34, and CD 45) and HLA-DR.

### A Rodent Model of Ischemic Stroke

All animal experiments followed guidelines of the Institutional Animal Care and Use Committee (IACUC140012) at CHA University. A model of transient middle cerebral artery occlusion (MCAo) was generated as described by Longa et al. (1989), using adult male Sprague–Dawley rats (Orient Bio, Seoul, South Korea) 270–300 g in weight. Once anesthesia was achieved by injecting ketamine (1%, 30 mg/kg) and xylazine hydrochloride (4 mg/kg) intraperitoneally, a ventral neck incision was made, exposing the right common carotid (CCA), external carotid (ECA), and internal carotid (ICA) arteries. In total, a 22-mm length of 4–0 monofilament nylon suture (PN.403756PK10; LMS Company, Seoul, Korea) was inserted through ECA into ICA lumen to block MCA at its origin for 90 min. This suture was then carefully removed. The rats were maintained at body temperatures of 37 ± 0.5°C using a heating pad (monitored by the rectal probe) and kept warm during recovery. To select appropriate stroke models, acute neurologic assessments (scored 0–5) of forelimb and hindlimb placement and appraisals of circling behavior took place 24 h after MCAo induction. Only those animals with scores ≤3 qualified for experimentation.

### IV Injection or IC Transplantation of hUC-MSCs

One week after MCAo induction (typically considered a subacute phase of stroke), hUC-MSCs were prepared for IV injection or IC transplantation by thawing stored cryovials in a water bath (37°C) within 1 min. For IV injection, a full cryovial was transferred to a 15-ml conical tube containing saline, gently swirled, and centrifuged at 1,000 rpm for 5 min. The supernatant was then aspirated, resuspending the pellet in saline. Doses of 1 × 10^6^ cells in 500 μl of saline (IV injection) or 1 × 10^6^ cells in 8 μl of saline (IC transplantation) were administered. Experimental groups were designated as follows: (1) IV saline only (500 μl) infused into tail veins (*n* = 13); (2) IC saline only (8 μl) delivered to ipsilateral hemispheres of the brain (*n* = 12); (3) IV hUC-MSCs (as above) infused into tail veins (*n* = 11); and (4) IC hUC-MSCs (as above) delivered to ipsilateral hemispheres of the brain (*n* = 11). Coordinates for stereotaxic injections of saline or hUC-MSCs were as follows (Tornero et al., 2013): anteroposterior (AP), +1.0 mm from Bregma; mediolateral (ML), −2.5 mm from midline; and dorsoventral (DV), −4.0/−7.0 mm from the surface. All transplanted rats were immunosuppressed through daily intraperitoneal injections of cyclosporine A (5 mg/kg/day; CKD Pharmaceutical Company, Seoul, Korea) starting 1 day before transplantation and continuing for up to 7 weeks post-transplantation.

Rats receiving IV-injected or IC-implanted hUC-MSCs were later sacrificed at 3 days, 4 weeks, and 7 weeks post-treatment for histologic examinations of cell survival within the brain (*n* = 5 at each time point per group).

### Behavioral Tests

Effects of administered hUC-MSCs were assessed by experimenters blinded to treatment status, performing stipulated behavioral tests [rotarod, stepping, and modified neurological severity score (mNSS)] weekly after MCAo for up to 8 weeks. In the rotarod test (Jeong et al., 2003), all rats are placed on a rotating rod set to progressively accelerate from 4 to 40 rpm in 120 s Times at which animals fell during rotation were recorded as the average of three trials.

For the stepping test (Olsson et al., 1995), rats were stationed on a tabletop in a forelimb stance at nearly 90° bodily orientation. Once they seemed relaxed, they were nudged forward along the tabletop, counting the number of forepaw placements when moved slowly in forehand and backhand directions across a distance of 90 cm over 5 s The right and left steps were separately counted. This test was always performed by the same operator, and the rats were familiarized with the experimenter’s grip before testing.

The mNSS is a standard test (Reglodi et al., 2003; Oh et al., 2015) during which we totaled motor (0–5), sensory (0–2), limb-placing (0–12), beam-balance (0–6), and abnormal movement (0–3) scores. A maximum of 28 points was possible in severe conditions, scores of 0 reflecting normal states. Injuries were subsequently graded by total scores as follows: mild, 1–9; moderate, 10–19; or severe, 20–28. To determine baseline levels, rotarod and stepping tests were performed 1–3 days before treatment (pre-test). We also calculated recovery rates by measuring the percentages of final scores at 8 weeks to baseline scores for each behavioral test.

### BrdU Injection

**Figure 1:**
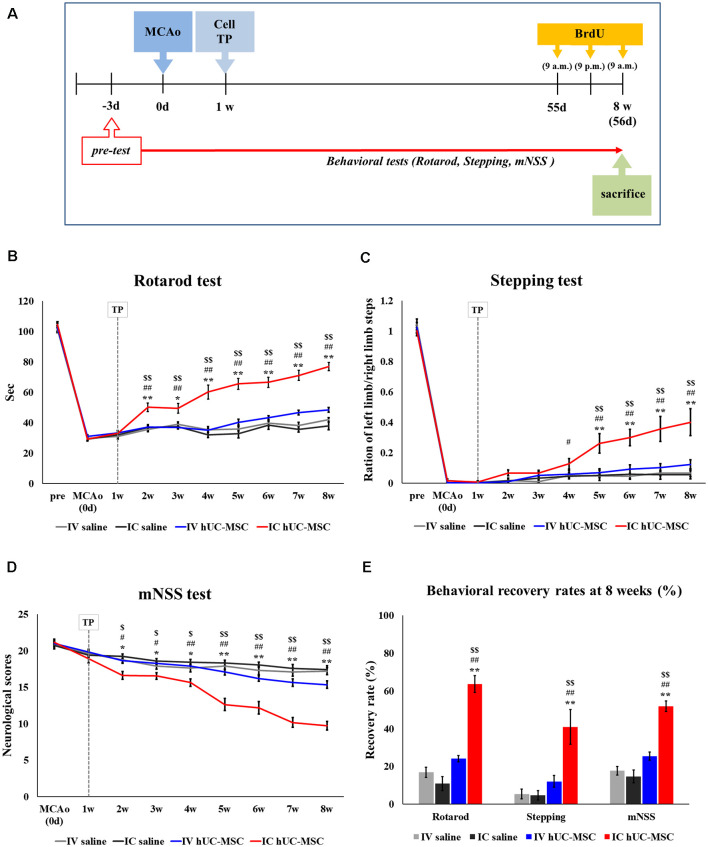
Behavioral tests after intracerebral (IC) transplantation or intravenous (IV) injection of human umbilical cord-derived mesenchymal stromal cells (hUC-MSCs) in middle cerebral artery occlusion (MCAo) rats. **(A)** Schematic of experimental designs. **(B)** Rotarod tests in four study groups for 8 weeks after treatment. **(C)** Stepping test for 8 weeks. **(D)** mNSS test for 8 weeks. **(E)** Behavioral recovery rates for three behavioral tests. The recovery rate is defined as the percentage of the final score (Week 8) to the baseline score (Day 0). Cell transplantation (“TP”) performed 1 week after MCAo induction. All data expressed as mean ± SEM values. *p < 0.05; **p < 0.001 (IV saline group vs. IC hUC-MSC group); ^#^*p* < 0.05; ^##^*p* < 0.001 (IC saline group vs. IC hUC-MSC group); ^$^*p* < 0.05; ^$$^*p* < 0.001 (IV hUC-MSC group vs. IC hUC-MSC group) by mixed ANOVA.

Immediately before the sacrifice of treated animals, 5′-Bromo-2′-deoxyuridine (BrdU, 50 mg/kg; Sigma–Aldrich) was injected intraperitoneally three times at 12-h intervals to detect endogenously proliferating cells (Figure 1A).

### Tissue Preparation

For immunohistochemical analysis, we sacrificed rats at 8 weeks post-MCAo, perfusing the hearts with saline and 4% paraformaldehyde (PFA). The brains were extracted and post-fixed overnight in 4% PFA at 4°C, then incubated in 30% sucrose at 4°C to equilibrate. Once frozen, 40-μm coronal sections were obtained by cryostat (CM3050S; Leica Microsystems, Wetzlar, Germany) for storage at −20°C. To measure infarct volumes, frozen coronal sections were cut in parallel at 16 μm.

### Immunohistochemistry (IHC)

Primary antibodies used for immunohistochemistry (IHC) were directed at the following antigens: glial fibrillary acidic protein (GFAP, 1:500; BD Biosciences, Franklin Lakes, NJ, USA), ionized calcium-binding adapter molecule-1 (Iba-1, 1:250; Wako Chemicals, Osaka, Japan), CD68 (ED1, 1:250; AbD Serotec, Oxford, UK), CD206 (1:200; Santa Cruz Biotechnology, Dallas, TX, USA), inducible nitric oxide synthase (iNOS, 1:200; Santa Cruz Biotechnology), BrdU (1:200; BD Biosciences), doublecortin (DCX, 1:250; Cell Signaling Technology, Danvers, MA, USA), proliferating cell nuclear antigen (PCNA, 1:200; Santa Cruz Biotechnology), polysialylated-neural cell adhesion molecule (PSA-NCAM, 1:200; Millipore Sigma, Burlington, MA, USA), RECA-1 (1:250; Abcam, Cambridge, UK), platelet-endothelial cell adhesion molecule-1 (PECAM-1, 1:200; R&D Systems, Minneapolis, MN, USA), and human nuclear antigen (hNu, 1:100; Millipore Sigma). Secondary antibody conjugates (1:250; Invitrogen, Carlsbad, CA, USA) included goat anti-mouse IgG/Alexa Fluor 488, goat anti-rabbit IgG/Alexa Fluor 488, goat anti-mouse IgG/Alexa Fluor 555, goat anti-rabbit IgG/Alexa Fluor 555, and donkey anti-goat IgG/Alexa Fluor 555. Staining patterns were examined and photographed using a laser-scanning confocal microscopy imaging system (LSM 510; Carl Zeiss Inc., Oberkochen, Germany).

### Determination of Infarct Volume

Eight coronal cryosections serially cut at 16 μm were stained with Cresyl violet in each group (*n* = 8 each). Ischemic and total hemispheric areas in each section were traced and measured, summing values of eight serial sections in the coronal plane for each brain. Based on intact contralateral hemispheres, infarct sizes were gauged as follows (Oh et al., 2015): estimated infarct size (%) = [1 − (area of remaining ipsilateral hemisphere/area of intact contralateral hemisphere)] × 100. Areas of interest were measured *via* ImageJ software (National Institutes of Health, Bethesda, MD, USA), summing values from eight serial coronal sections in each brain.

### Cell Counting

IHC quantitative analysis entailed cell counting in three coronal sections of the brain (AP: +1.5 mm, 0.0 mm, and −1.5 mm from Bregma), subjecting five rats of each group to ImageJ (NIH) particle analysis. To measure post-treatment host immune responses, numbers of ED1^+^, Iba-1^+^, CD206^+^, and iNOS^+^ cells (Sun et al., 2012; Ishizaka et al., 2013) were counted in five regions of interest (ROIs) along infarct border zones at 40× magnification by laser-scanning confocal microscope (Eclipse E600; Nikon, Tokyo, Japan). To quantify GFAP^+^ glial scarring (Bacigaluppi et al., 2009), GFAP^+^ areas in two representative ROIs of the peri-infarct cortex and striatum were measured at 20× magnification. Data were expressed as mean area (μm^2^)/ROI values. Extents of endogenous neurogenesis (Zhang et al., 2011) were determined by examining dual IHC markers for proliferating cells (BrdU and PCNA) or neuroblasts (DCX and PSA-NCAM), counting immunoreactive cells for each marker in three ROIs of ipsilateral subventricular zones (SVZs). Cerebrovascular ingrowth was similarly determined (Ukai et al., 2007; Ryu and McLarnon, 2009; Jantaratnotai et al., 2011; Ishizaka et al., 2013), counting diminutive (<30 μm) RECA-1^+^ cerebral vessels in four ROIs of peri-infarct border zones.

### Statistical Analysis

All computations relied on standard software (Enterprise v4.1; SAS Software, Cary, NC, USA), powered by the CHA University mainframe computer. Histologic parameters and infarct measurements were assessed by the Mann–Whitney *U* test, invoking a two-way analysis of variance (mixed-ANOVA) for behavioral data. Results were expressed as mean ± SEM (standard error of mean) values, setting significance at *p* < 0.05.

## Results

### Intracerebral Transplants of hUC-MSCs Ameliorate Neurologic Deficits in MCAo Rats

Effective IV doses of MSCs have ranged from 5 × 10^5^ to 3 × 10^6^ in rat models of stroke previously reported (Liu et al., 2014); and we have observed functional recovery in an acute model of stroke by infusing 1 × 10^6^ hUC-MSCs intravenously (Oh et al., 2018). Consequently, doses of 1 × 10^6^ cells were stipulated for the current experiment, assessing behavioral outcomes of IV injection and IC transplantation 8 weeks after MCAo induction.

Results of rotarod testing indicated significant improvement by Week 8 (vs. Week 1) in IC-treated rats, compared with IV-treated animals or those in either control group (IV or IC saline recipients; Figure 1B). Degrees of improvement gradually increased during the 7 weeks post-transplantation. In the stepping test, IC-treated rats also showed significant recovery between 3 and 7 weeks post-treatment, compared with IV-treated or control animals (Figure 1C); and mNSS assessments paralleled rotarod test results, the IC-treated rats demonstrating significant recovery between Week 1 and Week 7 weeks post-treatment, compared with IV-treated animals or those of either control group (Figure 1D). Collectively, final behavioral scores after IC transplantation of hUC-MSCs reflected significant improvement from baseline levels in all three testing categories, relative to scores achieved after IV-injection of hUC-MSCs or saline-only IV or IC delivery (Figure 1E).

Compared with controls, IV injections of hUC-MSCs yielded no functional improvement whatsoever by any behavioral measure 7 weeks after treatment. Hence, IC transplantation seemed preferable to IV injection for delivery of hUC-MSCs in our rodent model of subacute-phase ischemic stroke.

### Intracerebral Transplants of hUC-MSCs Reduce Infarct Volumes

**Figure 2:**
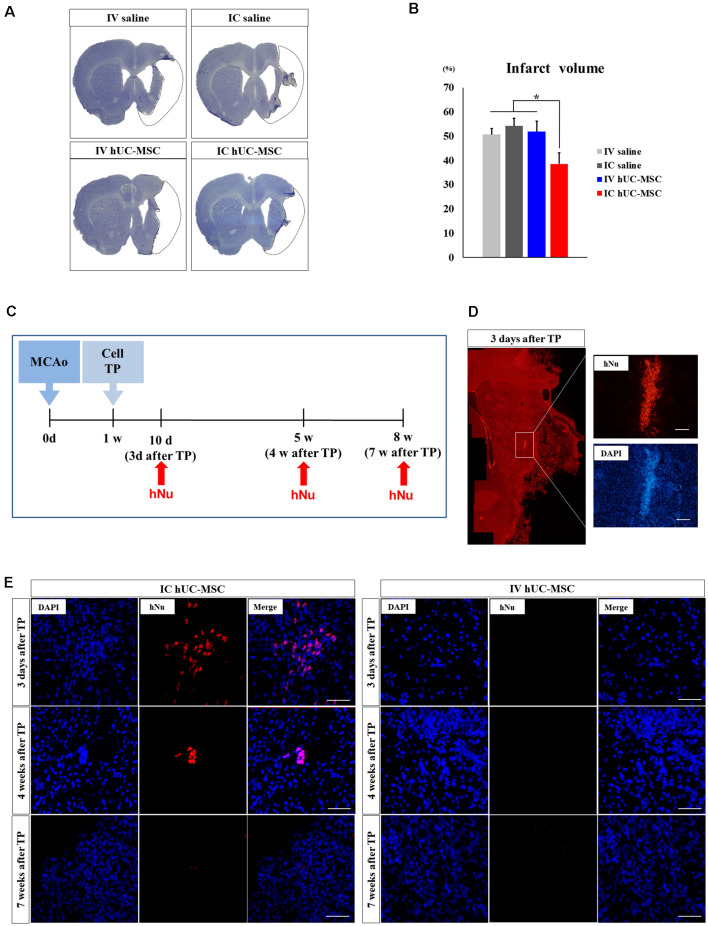
Comparison of infarct volumes and cell survival times in brain tissue after IC transplantation or IV injection of hUC-MSCs. **(A)** Cresyl violet staining in four study groups 8 weeks after MCAo induction. Black dashed line denotes arbitrary outline of infarct (scale bar = 100 μm). **(B)** Quantitative analyses of infarct volumes in four study groups (*n* = 8 each), data expressed as mean ± SEM values. *p < 0.05. **(C)** Schematic diagram of *in vivo* cell survival experiment (MCAo, point of middle cerebral arterial occlusion; Cell TP, point of cell or saline delivery in MCAo rats; hNu, point of hNu IHC). **(D,E)** Representative images of immunohistochemistry (IHC) for hNu at 3 days, 4 weeks, and 7 weeks after cell delivery. Note: hNu+ cells detected at 3 days and 4 weeks after IC transplants of hUC-MSCs; no hNu^+^ cells were detected after IV injection of hUC-MSCs. DAPI (blue) counterstain. Scale bar = 50 μm.

To determine how IC-transplanted hUC-MSCs impact neuronal injury after MCAo, we evaluated changes in infarct volumes by Cresyl violet staining of samples taken 7 weeks after treatment (Figure 2A). Mean infarct volume was significantly less in IC-treated rats (38.6 ± 4.5%) than in IV-treated animals (51.9 ± 4.3%) or in saline-only controls (IV, 50.6 ± 2.5%; IC, 54.2 ± 3.2%; Figure 2B). IV-treated rats did not differ from the two saline-only control groups in terms of infarct volume. These results suggest that a significant reduction in infarct volume is one therapeutic benefit of IC-transplanted hUC-MSCs, enabling functional recovery in MCAo rats.

### Survival of IC-Transplanted vs. IV-Injected hUC-MSCs Within Brain Tissue

We evaluated the survival of IV-injected and IC-implanted hUC-MSCs (Figure 2C), examining the hNu marker in three coronal sections of brain tissue (AP: +1.0, 0, −1.0 from the Bregma) obtained at 3 days, 4 weeks, and 7 weeks after treatment (*n* = 5 per group at each time point; Figure 2C). In rats given IC transplants of hUC-MSCs, striatal hNu^+^ cells were present 3 days after treatment (Figure 2D), although their numbers declined by 4 weeks, becoming undetectable at 7 weeks (Figure 2E). In rats given IV injections of hUC-MSCs, hNu^+^ cells were undetectable within in any regions of the brain at any time point (Figure 2E). This implies that IV-injected hUC-MSCs do not promote functional recovery, failing to migrate into the ischemic brain, whereas IC-transplanted hUC-MSCs (however short-lived) likely confer functional recovery through bystander effects of engrafted cells.

### Transplanted hUC-MSCs Modulate Inflammatory Responses in the Host Brain

**Figure 3:**
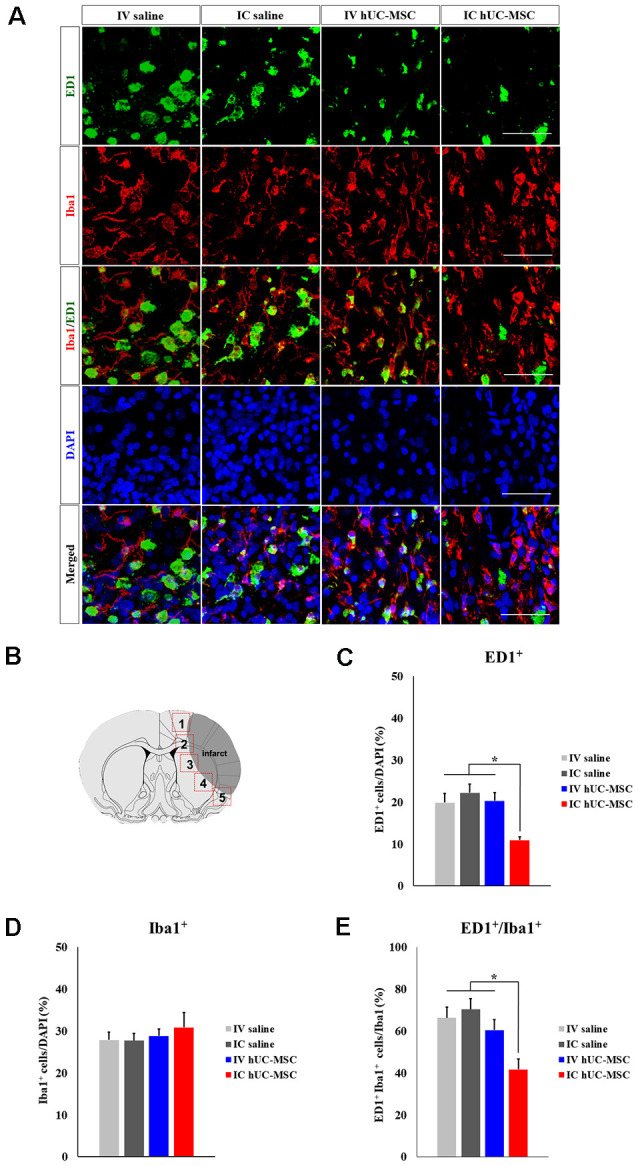
Microglial activation after IC transplantation or IV injection of hUC-MSCs in MCAo rats. **(A)** Representative confocal images of dual IHC for ED1 (green) and Iba-1 (red). DAPI (blue) counterstain. Scale bar = 50 μm. **(B)** Schematic images of quantitative analyses for five regions of interest (ROIs) in peri-infarct border zones. **(C)** Quantitative analyses of ED1^+^ cell proportionalities in four study groups (*n* = 5 each). **(D)** Quantitative analyses of Iba-1^+^ cell proportionalities in four study groups (*n* = 5 each). **(E)** Quantitative analyses of ED1^+^/Iba-1^+^ cell proportionalities in four study groups (*n* = 5 each). Data expressed as mean ± SEM values. *p < 0.05.

Human MSCs are known to modulate neuroinflammation in the damaged brain following cerebral ischemia (Iadecola and Anrather, 2011). To clarify the immunomodulatory roles of hUC-MSCs, we examined inflammatory responses within peri-infarct border zones of ipsilateral hemispheres (Figure 3A). Immunostained preparations of tissues bordering infarcts (Figure 3B) showed significantly fewer activated ED1^+^ microglia/macrophages in IC-treated rats, compared with IV-treated animals or saline-only controls (IV or IC delivery; Figure 3C), whereas cells positive for Iba-1 (a pan-microglia marker) did not differ quantitatively across the four groups studied (Figure 3D). Because Iba-1 is expressed by both resting and activated microglia, activated cells should display dual positivity (Iba-1^+^/ED1^+^; Bauer et al., 1994). Such cells were proportionately fewer in IC-treated rats (41.7 ± 5.0%), than in IV-treated animals (60.7 ± 5.0%) or in saline-only controls (IV, 60.5 ± 5.0%; IC, 70.3 ± 5.2%; Figure 3E).

**Figure 4:**
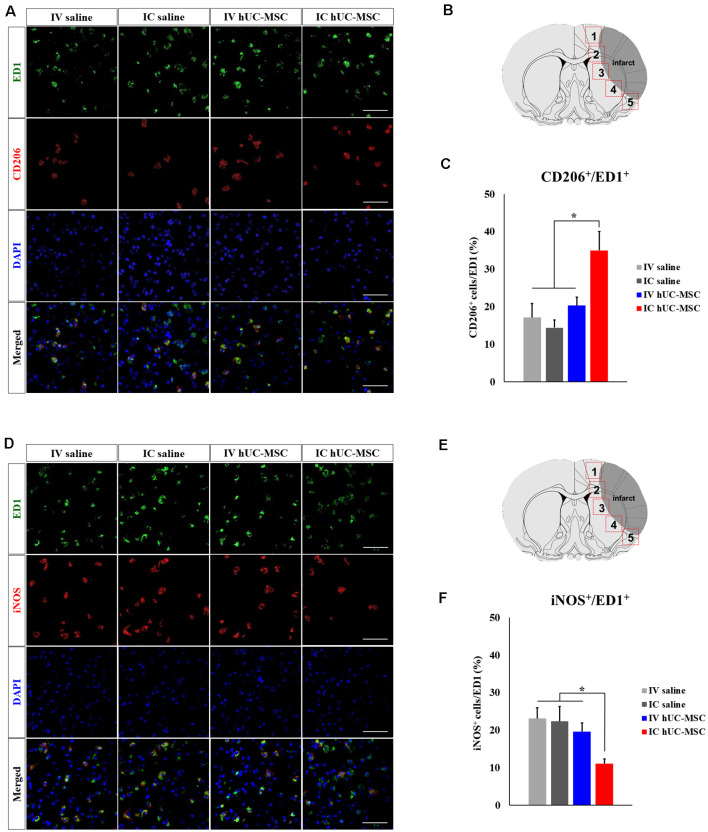
Microglial polarization after IC transplantation or IV injection of hUC-MSCs in MCAo rats. **(A)** Representative confocal images of dual IHC for ED1 (green) and CD206 (red). **(B)** Schematic images of quantitative analyses for five ROIs in peri-infarct border zones. **(C)** Quantitative analyses of ED1^+^/CD206^+^ cell proportionalities in four study groups (*n* = 5 each). **(D)** Representative confocal images of dual IHC for ED1 (green) and iNOS (red). DAPI (blue) counterstain. Scale bar = 50 μm. **(E)** Schematic images of quantitative analyses for five ROIs in peri-infarct border zones. **(F)** Quantitative analyses of ED1^+^/iNOS^+^ cell proportionalities in four study groups (*n* = 5 each). DAPI (blue) counterstain. Scale bar = 50 μm. Data expressed as mean ± SEM values. *p < 0.05.

We then investigated microglial polarization after IV injection or IC transplantation of hUC-MSCs. In dual-marker (CD206 and ED1) immunostaining of peri-infarct borders tissues (Figures 4A,B), CD206^+^/ED1^+^ cells were proportionately more numerous in IC-treated rats (35.0 ± 1.0%), compared with IV-injected animals (20.3 ± 2.2%) or either control group (IV, 17.1 ± 3.7%; IC, 14.4 ± 2.0%; Figure 4C). However, cells with dual iNOS^+^/ED1^+^ status in peri-infarct border zones (Figures 4D,E) were proportionately fewer in IC-treated rats (11.0 ± 1.2%), compared with IV-treated animals (19.6 ± 2.3%) or either control group (IV, 23.1 ± 2.8%; IC, 22.4 ± 3.9%; Figure 4F). Proportions of Iba-1^+^/ED1^+^ cells, CD206^+^/ED1^+^ cells, and iNOS^+^/ED1^+^ cells in IV-treated rats and in the two control groups were similar. The above findings strongly suggest that IC-transplanted hUC-MSCs may ameliorate neuroinflammation and promote healing of the ischemic brain.

### Transplants of hUC-MSCs Diminish Glial Scarring

**Figure 5:**
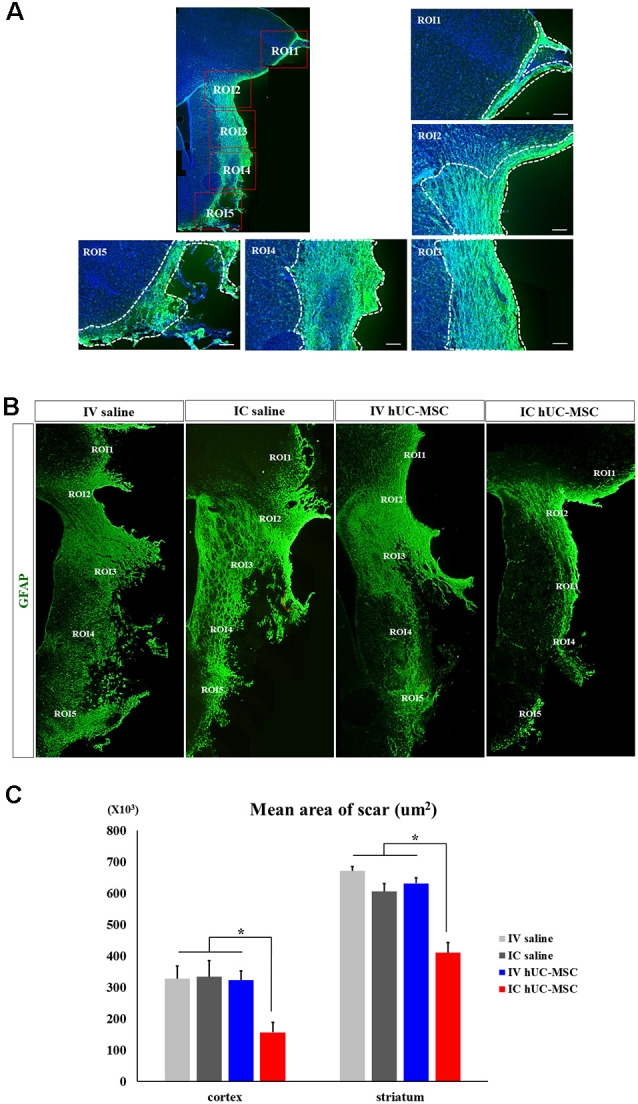
Glial scarring after IC transplantation or IV injection of hUC-MSCs in MCAo rats. **(A)** Schematic images of quantitative analyses for two ROIs in peri-infarct border zones. White dashed boxes denote ROIs for measuring GFAP^+^ glial scars within the peri-infarct cortex (i) and striatum (ii). Scale bar = 100 μm. **(B)** Representative confocal images of GFAP^+^ glial scars (green) in peri-infarct border zones of four study groups. **(C)** Quantitative analyses of GFAP+ glial scars in the peri-infarct cortex (left) and striatum (right) shown by four study groups (*n* = 5 each). Data expressed as mean ± SEM values. *p < 0.05.

Glial scar formation is among the major factors hampering patient recovery after ischemic strokes (Bacigaluppi et al., 2009). We investigated whether hUC-MSC delivery by different means might alter the extent of glial scarring. Within ipsilateral hemispheres, GFAP^+^ glial scars of cortex bordering infarcts (Figures 5A,B) were significantly less extensive in IC-treated rats (156.7 ± 31.1 μm^2^) than in IV-treated animals (417.7 ± 36.9 μm^2^) or in saline-only controls (IV, 327.9 ± 40.3 μm^2^; IC, 313.9 ± 77.6 μm^2^; Figure 5C, left panel). Results were similar within striatum (IC-treated rats, 273.0 ± 20.2 μm^2^; IV-treated rats, 417.7 ± 36.9; IV controls, 452.2 ± 18.1 μm^2^; IC controls, 430.2 ± 59.8 μm^2^; Figure 5C, right panel). IV-treated animals and the two saline-only control groups did not differ significantly in this regard, within cortex or striatum. It thus appears that IC-transplanted hUC-MSCs diminish glial scarring in peri-infarct border zones following cerebral ischemia.

### Transplants of hUC-MSCs Promote Endogenous Neurogenesis

**Figure 6:**
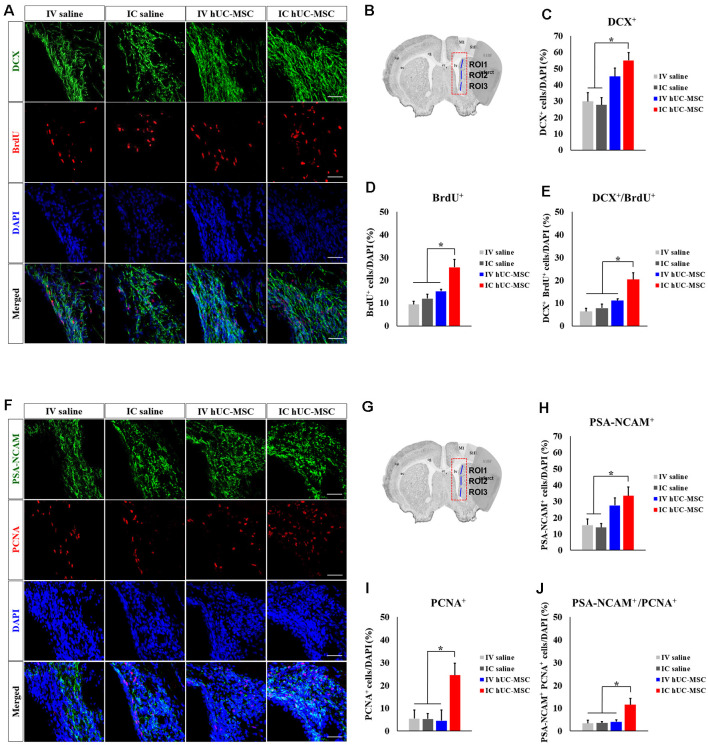
Subventricular zone (SVZ) neurogenesis after IC transplantation or IV injection of hUC-MSCs in MCAo rats. **(A)** Representative confocal images of dual IHC for DCX (green) and 50-Bromo-20-deoxyuridine (BrdU; red). **(B)** Schematic images of quantitative analyses for three ROIs in SVZs ipsilateral to infarcts. **(C)** Quantitative analyses of DCX^+^ cell proportionalities in four study groups (*n* = 5 each). **(D)** Quantitative analyses of BrdU^+^ cell proportionalities in four study groups (*n* = 5 each). **(E)** Quantitative analyses of DCX^+^/BrdU^+^ cell proportionalities in four study groups (*n* = 5 each). **(F)** Representative confocal images of dual IHC for PSA-NCAN (green) and PCNA (red). **(G)** Schematic images of quantitative analyses for three ROIs in SVZs ipsilateral to infarcts. **(H)** Quantitative analyses of PSA-NCAM^+^ cell proportionalities in four study groups (*n* = 5 each). **(I)** Quantitative analyses of PCNA^+^ cell proportionalities in four study groups (*n* = 5 each). **(J)** Quantitative analyses of PSA-NCAM^+^/PCNA^+^ cell proportionalities in four study groups (*n* = 5 each). DAPI (blue) counterstain. Scale bar = 50 μm. Data expressed as mean ± SEM values. *p < 0.05.

We evaluated the potential impact of hUC-MSC delivery mode on endogenous neurogenesis, staining for BrdU, and DCX in ipsilateral SVZs (Figures 6A,B). Compared with saline-only IV and IC control groups, numbers of DCX^+^ neuroblasts were significantly greater in IC-treated rats (Figure 6C) but proved similar in IV and IC treatment groups. BrdU^+^ cells proliferating within SVZs were also significantly increased in IC-treated rats, relative to IV-treated animals and saline-only control groups (Figure 6D); and those with dual markers (DCX^+^/BrdU^+^ cells) were proportionately more numerous in IC-treated rats (20.4 ± 2.9%), exceeding totals in IV-treated animals (11.2 ± 0.7%) or in either control group (IV, 6.3 ± 1.4%; IC, 7.8 ± 1.8%; Figure 6E).

To validate this finding, we stained for both PSA-NCAM and PCNA (Figures 6F,G). PSA-NCAM^+^ neuroblasts were significantly more numerous in IC-treated rats, compared with saline-only IV and IC groups (Figure 6H), again showing proportionate similarity to IV-treated animals. Proliferating PCNA^+^ cells in IC-treated rats were proportionately more numerous than in the other three groups (Figure 6I). Neuroblasts with dual markers (PSA-NCAM^+^/PCNA^+^ cells) were also more frequent in IC-treated rats (10.8 ± 2.8%), compared with IV-treated animals (2.7 ± 0.8%) or saline-only controls (IV, 3.4 ± 1.4%; IC, 3.2 ± 0.6%; Figure 6J). Proportions of DCX^+^/BrdU^+^ cells and PSA-NCAM^+^/PCNA^+^ cells in IV-treated animals and saline-only control groups did not differ. Overall, our findings provide compelling evidence that IC-transplanted hUC-MSCs promote endogenous neurogenesis within the SVZs of stroke-damaged rat brains.

### Transplants of hUC-MSCs Promote Angiogenesis

**Figure 7:**
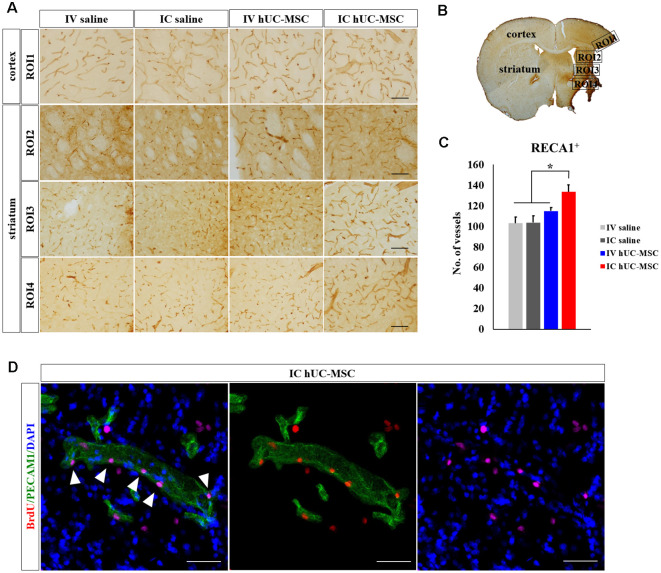
Peri-infarct angiogenesis after IC transplantation or IV injection of hUC-MSCs in MCAo rats. **(A)** Representative confocal images of DAB-stained RECA-1 (brown) in four study groups. Scale bar = 100 μm. **(B)** Schematic images of quantitative analyses for four ROIs in peri-infarct border zones. **(C)** Quantitative analyses of RECA-1^+^ microvascular (diameter < 30 μm) counts in four study groups (*n* = 5 each). **(D)** Representative confocal images of dual IHC for PECAM1 (green) and BrdU (red) in the rat after IC transplantation of hUC-MSCs. Note: No PECAM1^+^/BrdU^+^ vessels were found in the other three study groups. DAPI (blue) counterstain. Scale bar = 50 μm. Data expressed as mean ± SEM values. *p < 0.05.

Effects of hUC-MSC delivery modes on angiogenesis (i.e., new blood vessel formation), closely linked to neurogenesis, were assessed by measuring numbers of microcapillary vessels (<30 μm in diameter). RECA-1 immunostaining in peri-infarct border zones (Figures 7A,B) indicated significantly more microcapillary vessels in IC-treated rats (133.4 ± 6.8), compared with IV-treated animals (114.9 ± 3.3) or saline-only control groups (IV, 103.1 ± 6.0; IC, 103.6 ± 6.5; Figure 7C). The latter three groups did not differ in this regard. Dual staining of PECAM1 and BrdU was then pursued to measure actively proliferating peri-infarct blood vessels. Such cells were only detected in peri-infarct boundary zones of IC-treated rats (Figure 7D), attesting to the likelihood that IC-transplanted hUC-MSCs promote post-stroke angiogenesis in peri-infarct border zones.

## Discussion

To maximize the efficacy of transplanted hUC-MSCs, it is important to determine the optimal route and timing of administration, especially in dynamically changing environments of ischemic brain damage. The mode of stem-cell delivery is one issue critical for ensuring therapeutic efficacy in related clinical applications (Lindvall and Kokaia, 2011). Functional neural networks and cell-to-cell communication between grafted cells and the host brain are thus important prerequisites to achieving long-term stem-cell effects in this context.

Because the manner of MSC delivery largely determines cell survival and migratory capacity, we compared the therapeutic efficacy of two delivery modes (IV injection vs. IC transplantation) in a rodent model of subacute-phase stroke. Ultimately, we found that IC transplants of hUC-MSCs significantly improved functional outcomes, whereas IV injection fell short in most aspects. Behavioral recovery was readily observed, starting as soon as 1 week after delivery; and histologic improvements were durable, still observable at 7 weeks. Although our data and that of others (Roh et al., 2008; Li et al., 2016) underscore the short-lived nature of MSCs, the effects of cells that remain viable initially appear to persist, presumably through paracrine reverberations and cell-to-cell exchanges with host brain in terms of brain repair (Rodríguez-Frutos et al., 2016).

To date, IV injection of human MSCs (as opposed to IC transplants) has been favored in clinic settings, owing to the relative safety of this less invasive approach. However, the functional recovery achieved by IC transplantation of hUC-MSCs in subacute phases of stroke is lacking after IV injection, as our study shows. This failure reflects an inability to migrate and engraft within brain parenchyma after IV administration, documented in previous publications. Nearly 90% of IV-injected stem cells become trapped in the lungs, liver, or spleen, limiting their effects in treating brain injury (Walczak et al., 2008; Li et al., 2010; Pendharkar et al., 2010). Indeed, migration of circulating MSCs into brain parenchyma is perhaps more difficult in subacute (vs. acute) phases of injury, given the permanent damage to the blood-brain barrier and a dearth of chemotactic signals from the host brain. Another reason for migratory lapse may be the relatively low doses of injected cells that we used. Higher doses of MSCs (3 × 10^6^ cells) have produced histologic and behavioral improvements in one previous study (Lin et al., 2011). However, embolic risks (i.e., pulmonary embolism) are increased as doses rise, potentially aggravating hematologic and vascular instability in the aftermath of a stroke. Our observations suggest that IC transplantation is more effective than IV injection as a therapeutic strategy, requiring lower cell doses for therapeutic efficacy and preventing embolism at subacute-phases of stroke.

As we have demonstrated, IC-transplanted hUC-MSCs promote functional recovery through multiple mechanisms. They not only reduce pro-inflammatory responses and glial scarring but also spur SVZ neurogenesis and peri-infarct angiogenesis. These effects served in concert to confer neuroprotection from cerebral ischemia and impart subsequent functional recovery in stroke-damaged rat brains. In conditions of cerebral ischemia, inflammatory responses initiated by resident microglia and blood-borne macrophages persist for several weeks after ischemic insults (Jin et al., 2010). During the early stages of ischemic strokes (1–14 days), there is a proinflammatory shift in immune cells of the central nervous system, creating a prominent proinflammatory phenotype in rodent models of transient cerebral ischemia (Hu et al., 2012). A shift from pro-inflammatory to anti-inflammatory phenotype is activated during subacute phases of the stroke to dampen aberrant inflammation and commence healing of wounded brain tissues (Iadecola and Anrather, 2011). We found that IC transplantation of hUC-MSCs increased the proportion of anti-inflammatory cells (CD206^+^-ED1^+^ cells), relative to proinflammatory counterparts (iNOS^+^-ED1^+^ cells), thus reducing neuroinflammation and encouraging the processes of healing. IC-transplanted hUC-MSCs not only repair damaged brain tissue, chiefly through immunomodulation, but they also secrete trophic factors to recruit endogenous stem/precursor cells. These newly recruited cells may facilitate disease recovery and activate endogenous brain tissues by enhancing angiogenesis, neurogenesis, and synaptogenesis (Chopp and Li, 2002; Hsieh et al., 2010, 2013; Lin et al., 2011; Ernst et al., 2014; Frausin et al., 2015). As already reported, hUC-MSCs may simulate perivascular cells, secreting a multiplicity of proangiogenic cytokines (Choi et al., 2013) to promote angiogenesis in MCAo animal models (Toyama et al., 2009; Horie et al., 2011; Hsieh et al., 2013; Frausin et al., 2015).

Proliferating endothelial cells and new vessels continue to grow in the penumbra for at least 3 weeks after ischemic events (Hayashi et al., 2003). Concurrently, peri-infarct angiogenesis provides an instructive cue for SVZ neurogenesis through the molecular interplay between endothelial cells and neuroblasts (Ohab et al., 2006; Goldman and Chen, 2011). Human MSCs enhance SVZ neurogenesis by orchestrating a broad spectrum of neurotrophic factors (i.e., BDNF and GDNF; Ranganath et al., 2012). This proliferation of neural progenitors within the SVZ is accelerated during the first 2 weeks of the injury and lasts for several months (Arvidsson et al., 2002). Although we did not evaluate the ultimate fates of proliferating SVZ neuroblasts driven by IC transplants of hUC-MSCs, they are known to migrate into injured brain tissue to form mature neurons, thus restoring neural networks and leading to functional recovery (Arvidsson et al., 2002). Based on our reported findings, it is likely that endogenous brain repairs triggered by IC-transplanted hUC-MSCs begin early after treatment and yet are sustained for long periods.

In summary, present findings indicate that IC transplantation of MSCs is a more efficient means of delivering stem cells to target brain regions, reaping diverse functional benefits. Functional outcomes after cell-based therapy are route-dependent (Hao et al., 2014), requiring certain amounts of grafted cells to significantly attenuate functional deficits of ischemic injury. IC transplantation is particularly advantageous once a stroke has progressed to the subacute or chronic stage, whereas IV injection offers little therapeutic benefit. To bolster the survival of IC-transplanted MSCs in patients with strokes, the addition of trophic factors expressed by MSCs has been suggested (Steinberg et al., 2016). In future studies, biochemical and molecular analyses may also help elucidate the molecular mechanisms involved. However, we believe that the various behavioral and histologic improvements we have documented confirm the therapeutic utility of hUC-MSC transplantation in the endogenous repair of stroke-damaged brains.

## Data Availability Statement

The data that support the findings of this study are available from the corresponding author upon reasonable request.

## Ethics Statement

The animal studies were reviewed and approved by the CHA University Institutional Animal Care and Use Committee (IACUC140012). The human studies were approved by Institutional Review Board from CHA Bundang Medical Center for the use of human umbilical cord (hUC) samples (IRB no.: BD2013-004D).

## Author Contributions

J-EN: collection and assembly of data, data analysis, interpretation, and manuscript writing. S-HO and I-HP: data analysis and interpretation, and manuscript writing. JS: conception and design, financial support, administrative support, manuscript writing, and final approval of the manuscript. All authors contributed to the article and approved the submitted version.

## Conflict of Interest

JS is the founder and CEO of iPS Bio, Inc. The remaining authors declare that the research was conducted in the absence of any commercial or financial relationships that could be construed as a potential conflict of interest.
